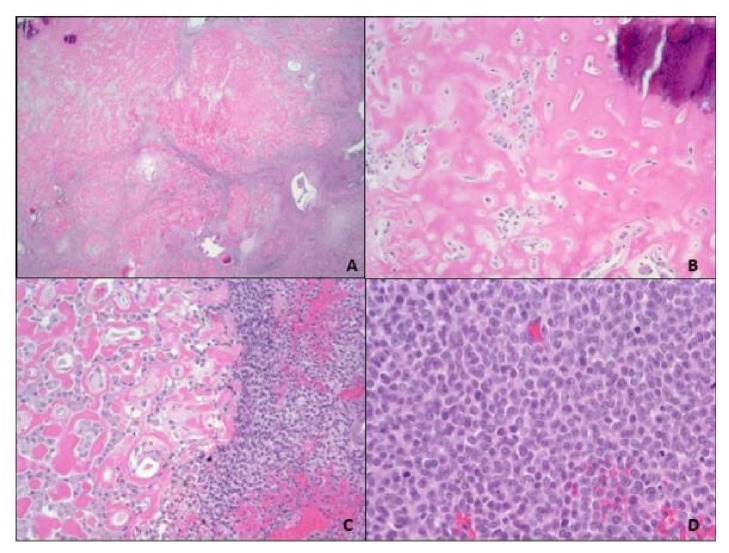# Ossifying Renal Tumor of Infancy: Laparoscopic Treatment and Literature Review

**DOI:** 10.1155/2018/1935657

**Published:** 2018-10-23

**Authors:** Ali Hajiran, Morris Jessop, Zachary Werner, Chad Crigger, John Barnard, Jeffrey Vos, Michael Ost

**Affiliations:** ^1^Suite 6300, Health Sciences Center Morgantown, WV 26506, USA; ^2^2144c HSCN Morgantown, WV 26506, USA

## Abstract

We present an unusual case of a rare ossifying renal tumor of infancy. A 6-month-old male initially presented with gross hematuria and without any palpable abdominal mass. Renal ultrasound and MRI showed a right lower pole, calcified, endophytic renal mass. Laparoscopic radical nephrectomy was performed without complications. Pathology demonstrated an ossifying renal tumor of infancy. We report this case, in addition to a review of the literature for similar cases, to highlight a rare renal tumor in infancy that can be managed laparoscopically.

## 1. Introduction

An ossifying renal tumor of infancy (ORTI) is an extremely rare finding, with only 24 published cases in the literature to date [1–3]. All reported cases have been unilateral and there have been no reports metastasis. The most common presenting symptom is gross hematuria, with only few patients presenting with a palpable abdominal mass on physical exam. Ultrasound (US), computed tomography (CT), and magnetic resonance imaging (MRI) have been used to characterize these tumors. Imaging typically shows a preserved renal outline with a calcified intrapelvic mass. Microscopically, these lesions show varying components of osteoid, osteoblastic cells, and spindle cells [4]. The treatment modalities for ossifying renal tumors of infancy are either nephrectomy or partial nephrectomy depending on the size and location of the tumor. A minimally invasive approach to solid renal tumors has become a universally accepted approach to treating renal masses in adult patients; however, laparoscopic renal surgery has yet to be routinely performed in the pediatric population, especially in children less than 12 months old. We report a case of a 6-month-old male with an ossifying renal tumor of infancy that was managed with a pure laparoscopic radical nephrectomy.

## 2. Case Report

**Figure 1:**
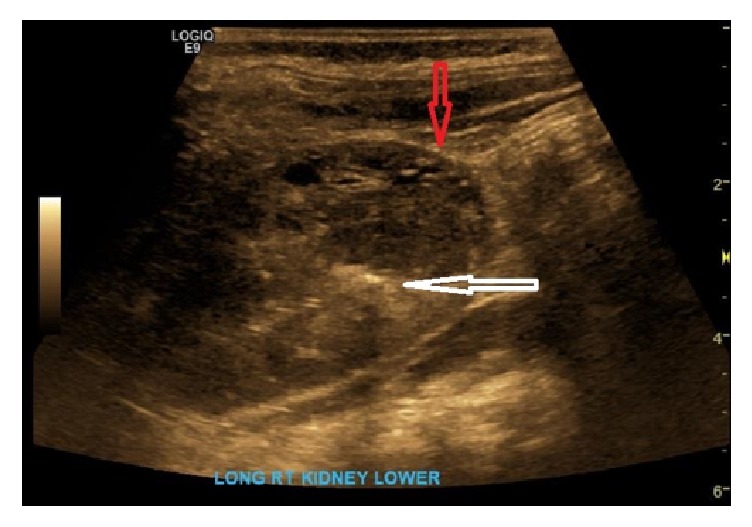


**Figure 2:**
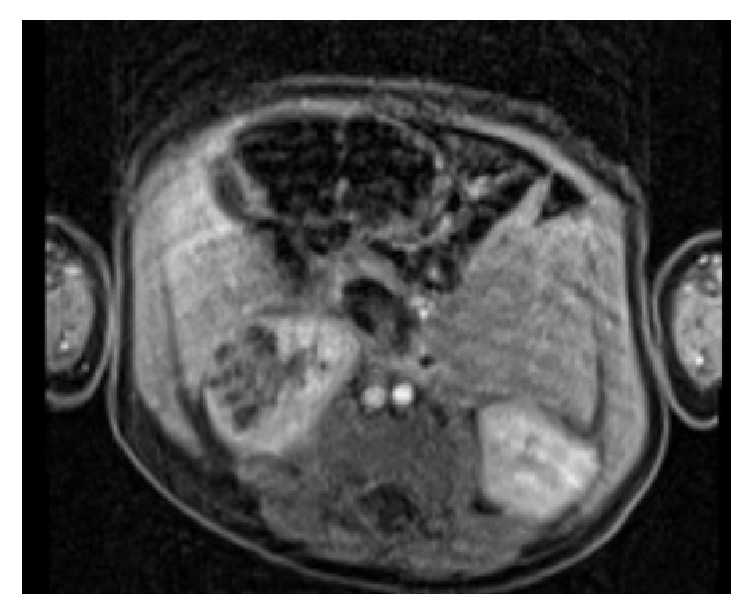


A 6-month-old male initially presented to his pediatrician to be evaluated for an episode of gross hematuria. The parents denied any history of fever, trauma, or any other associated symptoms. A renal ultrasound was performed which showed a right lower pole, predominantly solid, well-defined lesion with multiple small cystic components, measuring 3.2 x 1.8 x 2.6 cm, in addition to a small 2.1 mm linear calcification with shadowing (Figure 1). An MRI of the abdomen was performed with and without intravenous contrast that showed a right lower pole lesion with multiple T2 cystic components, measuring 2.8 x 2.5 x 1.8 cm (Figure 2). The cystic components were noted to be hypointense and nonenhancing on the postcontrast sequence with mild enhancement of the intervening septa.

Upon referral to our clinic, physical examination was unremarkable. The patient's abdomen was soft, nondistended, and nontender to palpation without any discernable palpable masses or hepatosplenomegaly. The patient's white blood cell count was 10,800/uL, hemoglobin was 12.0 g/dL, creatinine was 0.44 mg/dL, urinalysis was positive for blood, and urine culture was negative for infection. Hepatic function panel and electrolyte panel were within normal limits. The mass did not have hormonal function. A laparoscopic right radical nephrectomy was recommended.

**Figure 3:**
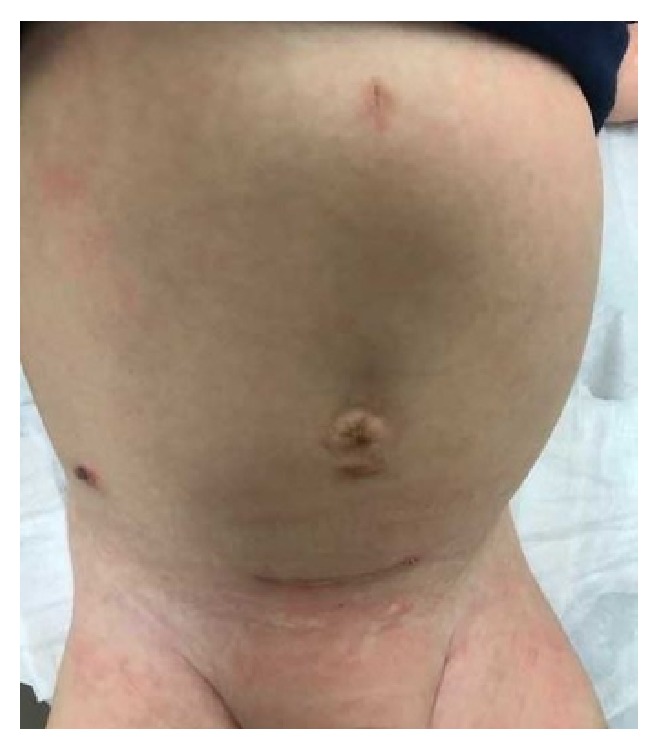


The patient was taken to the operating room and was given intravenous antibiotics for prophylaxis. After general anesthesia was induced, an orogastric tube and urinary catheter were inserted. The patient was placed into a modified left lateral decubitus position with the right flank up (Figure 3). Care was taken to pad all joints and the patient was secured to the operating table. A 5 mm port was placed at the umbilicus via open Hassan technique. Two other 5 mm working ports were placed under visualization in the left upper quadrant and subxiphoid. An additional left upper quadrant 5 mm port was placed for liver retraction.

First, the abdominal cavity was completely inspected. Next, the peritoneum was opened at the hepatic flexure outside of the colon. The colorenal ligaments were then incised over the kidney from the level of the liver down to the level of the inferior pole of the kidney. This allowed for complete reflection of the colon medially. We then began dissection inferiorly and medially and proceeded to skeletonize the ureter. The ureter was then tented up anteriorly and underneath the lower pole of the kidney. We proceeded to dissect out the renal hilum using a LigaSure™ device and suction. We skeletonized the hilum, which appeared to have a single artery and vein. We then created a plane in between the adrenal gland and the upper pole of the kidney. Using the LigaSure™ device, we took down the upper pole attachments, incising the hepatorenal ligaments all of the way to the lateral wall. The posterior attachments were also taken down using the LigaSure™ device. We proceeded to deploy a JustRight™ 5 mm device across the renal artery and vein en bloc.

**Figure 4:**
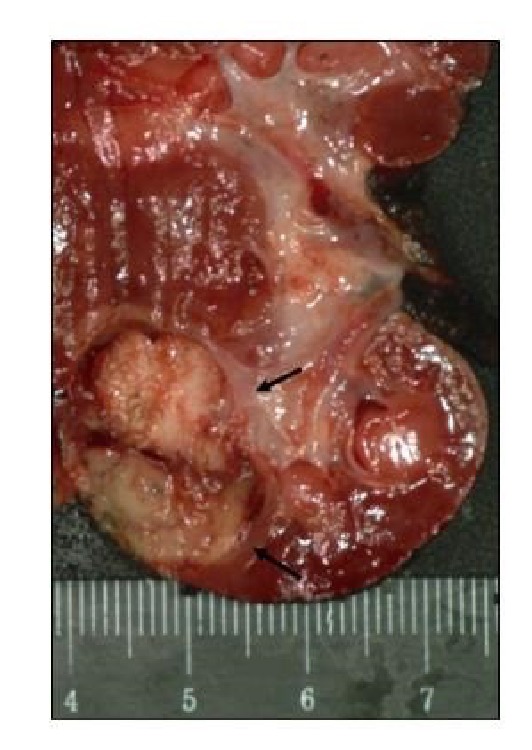


The kidney was freed in its entirety. We then proceeded to divide the ureter with the LigaSure™ device. Under vision we placed a 12 mm trocar along the right border of our previously marked Pfannenstiel incision in order to deploy a 10 mm EndoCatch™ bag. We then extended our incision medially along the Pfannenstiel mark, allowing us to remove the specimen intact in the EndoCatch™ bag. All fascial defects were closed (Figure 4).

**Figure 5:**
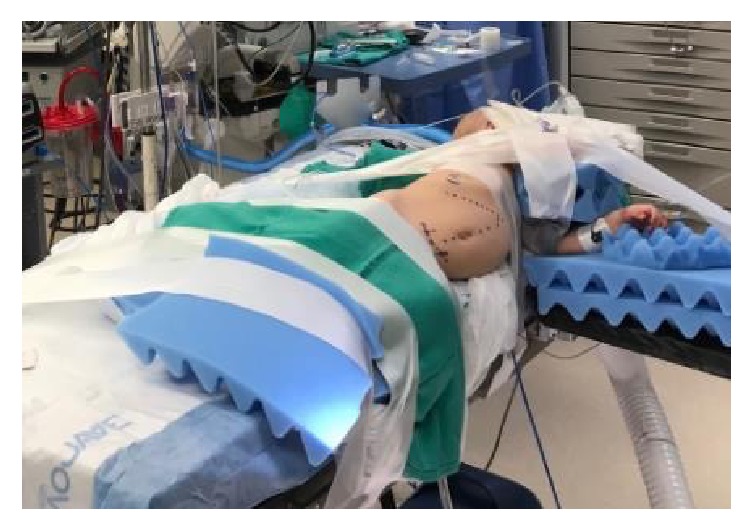


Pathologic macroscopic analysis of the specimen revealed a 1.4 x 1.3 x 0.8 cm white, indurated mass, 0.3 cm from the renal capsule (Figure 5). Microscopically the tumor was composed of an ossified core containing epithelioid cells with abundant cytoplasm surrounded by a spindle cell component with small, oval nuclei, and scattered mitoses (Figure 6). The tumor appeared well-circumscribed based on submitted sections. Margins were negative for invasion. The spindle cells showed moderate nuclear positivity for WT-1. Both populations were negative for AE1/3, desmin, synaptophysin, and CD99. While differential diagnosis included a blastemal predominant Wilms Tumor and congenital mesoblastic nephroma, the tumor was determined to be most consistent with a rare ossifying renal tumor of infancy.

## 3. Discussion

An ORTI is extremely rare. There are only 24 published cases in the literature since first reported by Chatten and colleagues in 1980 [1–3]. The average age at diagnosis is approximately 7 months, with the majority of cases diagnosed within the first 12 months of life [3]. These renal tumors are more common in males, with a 6:1 male-to-female ratio. All reported cases of ossifying renal tumors of infancy have been unilateral and there have been no reports of progression or malignant disease. The most common presenting symptom is gross hematuria, as seen in our patient. A palpable abdominal mass on physical exam is rare finding. In a review of 21 patients, 20 presented with gross hematuria (95%) and only 2 patients (10%) had a palpable abdominal mass on exam [3].

Preoperative diagnosis of an ossifying renal tumor of infancy can be challenging; however, authors have suggested that there are some characteristic imaging findings using ultrasound (US), computed tomography (CT), and magnetic resonance imaging (MRI). Ultrasonographic findings include a homogeneous, hyperechoic renal mass with posterior acoustic shadowing, and color Doppler demonstrating internal vascularity [5]. An ossifying renal tumor of infancy can be mistaken for a staghorn calculus due to its location within the collecting system and ossification [5–8]. CT scan typically reveals a preserved renal outline with an intrapelvic renal mass with calcifications seen on unenhanced imaging. In a review of 21 cases, 18 (85%) were found to have calcifications on preoperative imaging [3]. The 3 patients without calcifications in this series were diagnosed at younger ages, ranging from 6 days to 4 months old. ORTIs traditionally demonstrate poor enhancement on CT with intravenous contrast [8]. In regard to MRI, Lee and colleagues suggest that hypointensity on T2 weighted imaging is a unique feature of this tumor.

While our imaging findings clearly ruled out Wilms' Tumor (WT), it should be noted that WT was highly unlikely at the outset given age of presentation [4, 9]. Although WT is the most common renal malignancy of childhood, it most commonly presents as a palpable abdominal mass with a mean age of diagnosis of 3.2 years [9]. Microscopic hematuria, rather than gross hematuria, is more common upon presentation as well [9]. Congenital mesoblastic nephroma is the most common renal tumor in the newborn, with a mean age of presentation of less than 3 months and an association with polyhydramnios [10]. Although our patient's gestation did not have a history of polyhydramnios, this remained a possible diagnosis. ORTI should be suspected in infants presenting with gross hematuria; however diagnostic imaging is imperative for final differentiation amongst possible diseases.

Histologically, ORTIs are characterized by having varying components of osteoid, osteoblastic cells, and spindle cells [11]. Upon review of the pathology of nine cases, it was noted that the proportion of osteoid and degree of osseous maturation increased with increasing age of the patient. The authors of this series suggest that this tumor may represent an interaction between hyperplastic interlobular nephrogenic rests in the renal papilla with distal collecting ducts or urothelial cells in the developing kidney [11]. As these tumors arise from the renal papillae and extend into the collecting system, they can eventually lead to an obstructive process [1, 5, 11]. In regard to karyotype, clonal trisomy 4 has been reported as a characteristic finding using florescent in situ hybridization-probing [12]. In 2017, Vaillancourt and colleagues reported the first case of an ossifying renal tumor of infancy with positive WT-1 immunohistochemistry staining [2]. The authors warned of the possible misdiagnosis of a Wilms Tumor based on WT-1 positivity. The tumor in our patient also demonstrated moderate nuclear positivity for WT-1, which makes this the second report of this finding. This also suggests that ORTI, like multilocular cystic nephroma, is on the benign spectrum of Wilms Tumor.

ORTIs are believed to be benign in nature, as there have been no reported cases of metastatic spread. It is important to correctly diagnose this tumor because no adjuvant chemotherapy or radiotherapy is needed [7]. After 23 years of follow-up, a patient that underwent radical nephrectomy for an ossifying renal tumor of infancy at the age of 4 months was found to have no evidence of recurrence on imaging [13].

The treatment modalities for ossifying renal tumors of infancy are either nephrectomy or partial nephrectomy depending on the size and location of the tum [14]. In contrast to the vast majority of cases of Wilms' Tumor in which the appropriate surgical management involves radical nephrectomy, the benign nature of ORTIs makes them amenable to partial nephrectomies. Indeed, various authors have reported good outcomes in cases of ORTI with partial nephrectomies as shown in Hu and colleagues' review of the literature [14]. When surgically feasible, a nephron sparing surgery can provide adequate surgical outcomes. In children with solid renal tumors, an open surgical approach using a large transverse abdominal incision has traditionally been used [12]. In adult patients with renal tumors, multi-institutional studies have proved the efficacy and safety of laparoscopic nephrectomy; however, there remains a lack of prospective multicenter data regarding minimally invasive approaches in the pediatric literature [15–17]. In case reports and small retrospective series, several authors have reported that it is possible to perform laparoscopic nephrectomies safely in children and that this approach can be associated with reduced analgesic requirements, decreased length of stay, and equivalent outcomes compared to open nephrectomy [16, 18–21]. In 2018, Harris and colleagues reported a series of 43 pediatric patients (ages 0-10) who underwent laparoscopic versus open nephrectomy and summarized that a laparoscopic approach could be safely considered in pediatric renal tumors that do not cross the midline, with no signs of preoperative tumor rupture or local spread, provided that there is enough space to maneuver the tumor into an appropriately sized specimen bag [16].

## 4. Conclusion

An ossifying renal tumor of infancy, although extremely rare, should be considered in the differential diagnosis when evaluating pediatric patient with gross hematuria and an endophytic calcified cystic renal mass. Depending on the tumor's size and location and the surgeon's experience, a pure laparoscopic approach to radical nephrectomy can be considered in pediatric patients less than 12 months of age.

## Figures and Tables

**Figure 6:**